# Antibiotic prescribing in UK care homes 2016–2017: retrospective cohort study of linked data

**DOI:** 10.1186/s12913-020-05422-z

**Published:** 2020-06-18

**Authors:** Catherine M. Smith, Haydn Williams, Arnoupe Jhass, Selina Patel, Elise Crayton, Fabiana Lorencatto, Susan Michie, Andrew C. Hayward, Laura J. Shallcross, on behalf of the Preserving Antibiotics through Safe Stewardship group, N. Anderson, L. Atkins, A. Conolly, E. Crayton, S. Denaxas, P. Dutey-Magni, N. Elsay, G. Forbes, E. B. Fragaszy, N. Freemantle, C. Fuller, M. Gill, A. H. Hayward, R. Horne, A. Jhass, P. Kostkova, F. Lorencatto, S. Michie, J. Mindell, M. Richardson, J. Robson, P. Rockenschaub, C. Royston, L. J. Shallcross, C. M. Smith, E. Sutton, J. Thomas, C. Tarrant, R. Traina, E. Richardson, J. West, H. Williams

**Affiliations:** 1 Institute of Health Informatics, University College London, London, NW1 2DA UK; 2 Four Seasons Health Care, Norcliffe House, Station Road, Wilmslow, Cheshire, SK9 1BU UK; 3 Research Department of Primary Care and Population Health, University College London, London, NW3 2PF UK; 4 Centre for Behaviour Change, University College London, WC1E 7HB, London, UK; 5 Institute of Epidemiology and Health Care, University College London, WC1E 7HB, London, UK

**Keywords:** Anti-bacterial agents, Long-term care, Antibiotic stewardship, Antibiotic prescribing, care home, Older people, Infection

## Abstract

**Background:**

Older people living in care homes are particularly susceptible to infections and antibiotics are therefore used frequently for this population. However, there is limited information on antibiotic prescribing in this setting. This study aimed to investigate the frequency, patterns and risk factors for antibiotic prescribing in a large chain of UK care homes.

**Methods:**

Retrospective cohort study of administrative data from a large chain of UK care homes (resident and care home-level) linked to individual-level pharmacy data. Residents aged 65 years or older between 1 January 2016 and 31 December 2017 were included. Antibiotics were classified by type and as new or repeated prescriptions. Rates of antibiotic prescribing were calculated and modelled using multilevel negative binomial regression.

**Results:**

13,487 residents of 135 homes were included. The median age was 85; 63% residents were female. 28,689 antibiotic prescriptions were dispensed, the majority were penicillins (11,327, 39%), sulfonamides and trimethoprim (5818, 20%), or other antibacterials (4665, 16%). 8433 (30%) were repeat prescriptions. The crude rate of antibiotic prescriptions was 2.68 per resident year (95% confidence interval (CI) 2.64–2.71). Increased antibiotic prescribing was associated with residents requiring more medical assistance (adjusted incidence rate ratio for nursing opposed to residential care 1.21, 95% CI 1.13–1.30). Prescribing rates varied widely by care home but there were no significant associations with the care home-level characteristics available in routine data.

**Conclusions:**

Rates of antibiotic prescribing in care homes are high and there is substantial variation between homes. Further research is needed to understand the drivers of this variation to enable development of effective stewardship approaches that target the influences of prescribing.

## Background

Around one in seven people aged over 85 live in approximately 20,000 care homes in the United Kingdom [1–5]. This includes residential homes, which provide accommodation and personal care, and nursing homes, in which at least one qualified nurse is always on duty [6]. Care home residents are at increased risk of acquiring infections owing to age-related biological factors combined with environmental factors of the care home setting [7]. Chest infections, gastrointestinal infections, urinary tract infections, and skin and soft tissue infections are commonly reported and can cause outbreaks [7, 8].

As a result of the high frequency of symptoms that may indicate infection, antibiotics are used frequently for care home residents and there is potential for development of antibiotic resistance [9, 10]. Frequent antibiotic use can be problematic for this population because they are at increased risk of adverse events related to antibiotic treatment such as infection with *Clostridium difficile*, side effects, and drug-drug interactions [9]. Residents admitted to hospital frequently return to care homes and then go back to hospital, creating the opportunity for transmission of infections, including drug-resistant pathogens, between healthcare settings [11]. Avoiding these adverse events requires identification of opportunities to safely reduce antibiotic use (antibiotic stewardship). To do this, detailed information on how antibiotics are currently used in this setting is needed.

Current evidence on antibiotic prescribing in care homes in the UK has largely been derived from point-prevalence surveys, including three European Centre for Disease Prevention and Control (ECDC)-coordinated projects (Healthcare-associated infections in long-term care facilities, HALT) in 2010, 2013 and 2016–17 [12–14]. The most recent HALT survey involved care homes in 26 countries including 70 homes in Northern Ireland, 52 in Scotland and 28 in Wales (England did not participate). The point prevalence of antibiotic use was 4.9% across Europe and ranged from 5 to 10% in participating UK administrations [14]. A separate UK point prevalence survey, conducted in 644 long-term care facilities in 2017, found mean antibiotic prevalence of 7.7% in nursing homes and 6.7% in residential homes, and a mean of 1.04 antibiotics per resident [15]. Although these surveys provide overall estimates of antibiotic use, they are prone to seasonal variation and may not be representative.

A recent study described antibiotic prescriptions dispensed mainly to care home residents from a UK national pharmacy chain [16]. Although this study reported that half the residents included were prescribed at least one antibiotic per year, it did not include any resident data (such as age, date of entry to or exit from the care home, or date of death), precluding calculation of person-time denominators. The study was therefore unable to estimate rates of prescribing or assess factors associated with high prescribing. Analyses of primary care electronic health records have shown that prescribing increases in older age groups in the UK [17, 18]. However, these analyses provide little insight into patterns of prescribing to care home residents because care home residency is poorly recorded in UK electronic health records.

In this study, we used linked pharmacy and administrative data to investigate antibiotic prescribing to residents of a large chain of UK care homes. Our aims were to describe the types of antibiotics used for care home residents, estimate the rate of antibiotic prescribing, measure variation in prescribing by care home, and investigate care home and resident factors associated with prescribing.

## Methods

There is no mandatory surveillance system for care homes in the UK and care home residency is not routinely recorded in electronic health records (primary or secondary care). We therefore used administrative systems from a large chain of care homes and did a retrospective cohort study including residents of these homes between 1 January 2016 and 31 December 2017. Here we describe the data available in these systems. Care home administrative systems were used to describe the characteristics of residents and care homes. Individual-level prescriptions dispensed to residents of these homes were obtained by linkage to data from a national pharmacy chain.

### Care home administrative data

We extracted data from routine care home administrative systems on resident and care home characteristics. Resident characteristics available were: age, sex, length of stay, residential or nursing care (i.e. at least one qualified nurse on duty at all times), whether the resident had dementia, and their status at the end of the study period (in the home, transferred out, or died). Care home-level characteristics available were: location, number of beds, number of clinical and care staff, and Care Quality Commission (CQC) rating (homes in England only)). We also extracted information on suspected incidents of infection (number and type) for each resident reported during the study period through an internal incident monitoring system used by the care home chain. This system is not linked to microbiological testing and therefore does not differentiate between suspected infections (identified by care home staff) and those confirmed microbiologically. We classified homes as urban or rural and according to deprivation decile using country-specific indices [19–25].

### Dispensed prescriptions

A large national pharmacy chain is contracted to fulfil drug prescriptions for residents of the care home chain. We extracted British National Formulary (BNF) drug classifications and date dispensed from the pharmacy database on all drugs dispensed to residents of the care homes during the study period. We linked individual-level pseudonymised pharmacy and resident data using an identifier comprised of resident first initial, Soundex (a phonetic algorithm for indexing family names by sound), birth year, and care home identifier. Although the pharmacy chain is contracted to dispense all drugs to residents of the care home chain, we found that some care homes had few residents matching to prescriptions (suggesting that prescriptions were dispensed from elsewhere). We therefore excluded data from homes for which less than 75% of the residents had at least one prescription (of any drug or device). As a sensitivity analysis, we included only residents for which at least one prescription was matched (regardless of overall home proportion). In both analyses, we also excluded residents aged under 65 and individuals who entered and left the care home on the same day.

This study was approved by the UCL Research Ethics Committee (ID 11813/002).

### Description of residents, care homes and antibiotics

We described resident and care home-level characteristics using counts and proportions. As a simple measure of co-morbidity, we calculated the number of chapters of the BNF (excluding antibiotics) from which residents had repeated drug prescriptions during the study period. We assumed that repeated prescriptions from more chapters would indicate a likely higher level of co-morbidity (although this is not intended to represent an estimate of the number of conditions).

We identified antibiotics in the pharmacy data using BNF chapter 5 (infections) and subchapter 5.1 (antibacterials) [26, 27], and described antibiotic prescriptions by class using the Anatomical Therapeutic Chemical (ATC) system [28]. We classified antibiotics as new or repeated prescriptions using a cut-off of 35 days: If an antibiotic was prescribed within 35 days of a previous prescription of the same drug, it was classified as a repeat prescription. The 35 day cut-off was based on the distribution of time between prescriptions of the same antibiotic (Additional file 1). We calculated the total number of new and repeat prescriptions and the median number of repeats for each type of antibiotic.

### Estimation of antibiotic prescribing rates and factors associated with prescribing

We described resident and care home-level characteristics using counts and proportions. We calculated the crude rate of antibiotic prescribing per resident year and the median rate for individual residents. We plotted rates by care home and calculated the intra-class correlation coefficient.

We used multilevel negative binomial regression to model antibiotic prescribing rates including random effects to account for clustering at the level of the care home. We assessed single variable associations with resident- and care home-level variables and included variables that showed some association with the outcome in a multivariable model (age and gender were also included in multivariable models). Rural-urban classification, deprivation decile and CQC rating are not standardised across UK administrations. We therefore ran separate single- and multivariable models for England, Northern Ireland, Scotland, and Wales including these variables in addition to those in the main regression analysis.

Analyses were conducted using R v3.5.1, using the lme4 package for mixed effects models [29].

## Results

### Study population

Between 1 January 2016 and 31 December 2017, there were 27,075 residents of the 258 care homes operated by the chain. In 135 care homes at least 75% of residents matched to at least one pharmacy record, with a total of 14,194 residents in these homes. A further 657 residents were excluded because they were aged under 65 years and 50 because they entered and left the home on the same day. The cohort therefore included 13,487 residents of 135 care homes, who stayed for a total of 3,916,931 resident-days (10,731 resident-years) during the study period. Characteristics of residents and care homes included and excluded from the main analysis were similar (Additional file 2).

Resident and care home characteristics are shown in Table 1. Most care homes were in England (93/135, 69%), and located in urban areas (84/93, 90% care homes in England; 17/20, 85% in Northern Ireland; 19/19, 100% in Scotland, and 1/3, 33% in Wales). The median number of beds was 50 (range 25 to 111). The majority of residents were female (8518/13,487, 63%) and the median age was 85 (interquartile range (IQR) 79 to 90). Most residents had nursing care (9109/13,487, 68%), 39% (4217/13,487) had dementia, and 7027 residents (52%) died during the study period. The median number of BNF chapters from which residents had repeated prescriptions (excluding antibiotics) was 4 (IQR 1 to 5). The median resident length of stay during the study period was 210 days (IQR 51 to 509) and the median total resident length of stay (from date of admission to the home) was 333 days (IQR 67 to 913).

**Table 1:** Resident and care home characteristics

Variable	Number	Percentage
**Resident-level (*n*** **= 13,487)**		
Gender		
Male	8518	63.2
Female	4969	36.8
Age		
65–74	1871	13.9
75–84	5049	37.4
85–94	5691	42.2
95+	876	6.5
Type of care		
Residential	4354	32.3
Nursing	9109	67.5
Dementia		
No	8246	61.1
Yes	5217	38.7
Respite care		
No	11,459	85.0
Yes	2028	15.0
Length of stay during study period (days) Median (IQR)	210	51–509
Overall length of stay (days) Median (IQR)	333	67–913
Entered care home during study period		
No	5587	41.4
Yes	7900	58.6
Status at end of study period		
In home	3772	28.0
Permanently Discharged	2688	19.9
Died	7027	52.1
Number of reported infection episodes during study		
0	8993	66.7
1	2217	16.4
More than 1	2277	16.9
Number of BNF chapters with repeated prescriptions (excluding antibiotics)		
0–1	3907	29.0
2–4	4275	31.7
5–7	4693	34.8
8 or more	612	4.5
**Care home-level (*n*** **= 135)**		
Country		
England	93	68.9
Northern Ireland	20	14.8
Scotland	19	14.1
Wales	3	2.2
Number of beds		
< 40	34	25.2
40–49	37	27.4
50–59	34	25.2
60+	30	22.2
Median overall length of stay		
< 1 year	34	25.2
1–2 years	85	63.0
> 2 years	16	12.9
Clinical staff per 100 residents		
< 10	35	25.9
10–19	65	48.2
20+	35	25.9
Care staff per 100 residents		
< 60	33	24.4
60–79	75	55.6
80+	27	20.0
Percentage residents with dementia		
< 10	35	25.9
10–80	80	59.3
80–100	20	14.8
Percentage residents with nursing care		
< 10	20	14.8
10–80	58	43.0
80–100	57	42.2
Number of infection incidents per bed per year		
Less than 1	70	51.9
1 to 2	39	28.9
2 or more	26	19.3

### Antibiotics

**Fig. 1:**
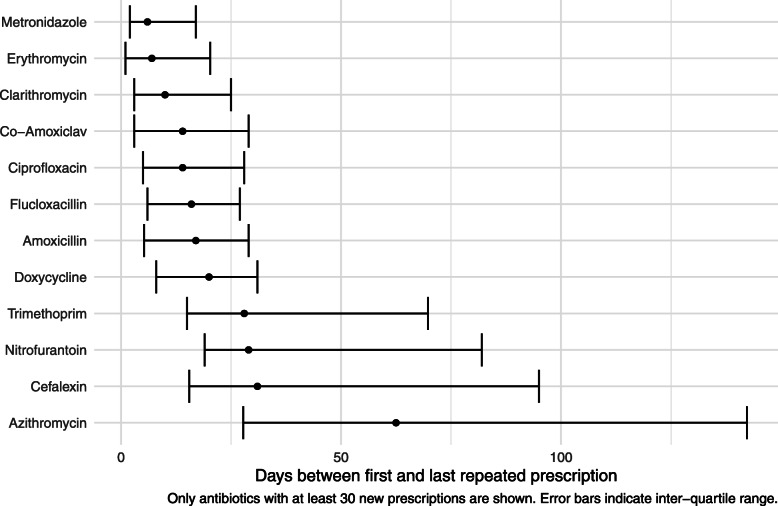
Length of repeated antibiotic prescriptions

A total of 28,689 antibiotic prescriptions were dispensed, and the most common ATC classes of antibiotics used were penicillins (11,327/28,689, 39% prescriptions), sulfonamides and trimethoprim (5818/28,689, 20%), and other antibacterials (4665/28,689, 16%). We classified 70% (20,223/28,689) antibiotics as new prescriptions and 30% (8466/28,689) as repeats. The prescription was a one-off for 89% (18,002/20,223) new prescriptions, the remaining 11% (2221/20,223) were new prescriptions that led to repeats. When the antibiotic was repeated at least once, the median number of antibiotic prescriptions was 2 (IQR 2–3, maximum 26), and the median number of days between the first and last prescription was 21 (IQR 8–35). The antibiotics that were repeated over the longest time were azithromycin, cephalexin, nitrofurantoin and trimethoprim (Fig. 1). The median number of days between the first and last prescriptions of azithromycin was 62 (IQR 28–142).

### Antibiotic prescribing rates and factors associated with prescribing

**Fig. 2:**
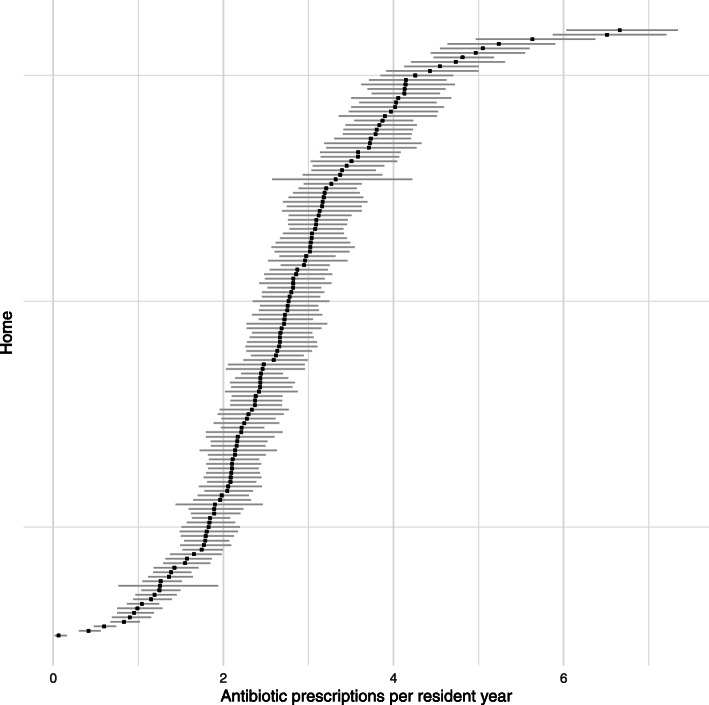
Crude rates and 95% confidence intervals of antibiotic prescribing by care home

The crude rate of prescribing was 2.68 prescriptions per resident year (95% confidence interval (CI) 2.64–2.71). The median rate of antibiotic prescriptions for an individual resident per year was 0.71 (IQR 0–3.70). Rates varied by home (median 2.67 IQR 2.07–3.29 antibiotic prescriptions per resident year, Fig. 2), but prescribing rates within homes were not highly correlated (intra-class correlation coefficient 0.19).

Rates of prescribing according to resident and care home characteristics and unadjusted results of negative binomial regression analyses are shown in Table 2 (country specific-models in Additional File 3). The final model (Table 3) was adjusted for resident age, sex, and variables that were associated with antibiotic prescribing at single variable analysis: residential or nursing care, dementia, respite care, care home entry during study period, status at end of study period (still in home, died, transferred out), number of infections reported, number of BNF chapters with repeat prescriptions. In the adjusted model, increased antibiotic prescribing was associated with care home entry during the study period (adjusted incidence rate ratio, aIRR 1.37, 95% CI 1.30–1.44); having nursing care (aIRR 1.21, 95% CI 1.13–1.30); death during the study period (aIRR 1.58, 95% CI 1.50–1.67), and those who were permanently discharged from the home (aIRR 1.44, 95% CI 1.32–1.57). Increased numbers of reported infections (aIRR for two or more infections compared to none 2.09, 95% CI 1.96–2.24) and higher levels of co-morbidity (aIRR for 5–7 BNF chapters with repeat prescriptions compared to 1 chapter 2.38, 95% CI 2.16–2.62; for 8 or more chapters 2.89, 95% CI 2.54–3.28) were also associated with increased antibiotic prescribing. There were no clear associations between antibiotic prescribing and resident age, sex, or care home-level variables.

**Table 2:** Rates and single variable analysis of antibiotic prescribing by resident and care home characteristics

Variable	Number of antibiotic prescriptions	Resident years	Antibiotic prescriptions per resident year	Incidence rate ratio (95% CI)
**Resident-level**				
Gender				
Male	9200	3518	2.61	Ref.
Female	19,489	7213	2.70	1.00 (0.95–1.06)
Age				
65–74	3823	1507	2.54	Ref.
75–84	10,159	3955	2.57	1.02 (0.94–1.10)
85–94	12,649	4556	2.78	1.08 (1.00–1.17)
95+	2058	713	2.89	1.16 (1.03–1.30)
Type of care				
Residential	8539	3434	2.49	Ref.
Nursing	20,071	7265	2.76	1.26 (1.17–1.35)
Dementia				
No	17,325	5996	2.89	Ref.
Yes	11,285	4703	2.40	0.86 (0.81–0.92)
Respite care				
No	27,461	10,184	2.70	Ref.
Yes	1228	547	2.24	0.89 (0.80–0.98)
Entered care home during study period				
No	17,190	6742	2.55	Ref.
Yes	11,499	3989	2.88	1.11 (1.06–1.17)
Status at end of study period				
In home	10,573	4599	2.30	Ref.
Permanently Discharged	2340	906	2.58	1.14 (1.05–1.24)
Died	15,776	5226	3.02	1.47 (1.39–1.55)
Number of reported infection episodes during study				
0	10,463	5405	1.94	Ref.
1	5654	2206	2.56	1.52 (1.42–1.62)
More than 1	12,572	3121	4.03	2.22 (2.07–2.37)
Number of BNF chapters with repeated prescriptions (excluding antibiotics)				
0–1	852	1032	0.83	Ref.
2–4	7971	3603	2.21	1.96 (1.77–2.16)
5–7	16,390	5267	3.11	2.48 (2.25–2.73)
8 or more	3476	829	4.19	3.19 (2.81–3.62)
**Care home-level**				
Country				
England	19,206	7441	2.58	Ref.
Northern Ireland	3971	1313	3.02	1.20 (0.94–1.52)
Scotland	4526	1726	2.62	1.04 (0.82–1.31)
Wales	986	251	3.93	1.72 (0.99–3.00)
Number of beds				
< 40	5708	1814	3.15	Ref.
40–49	6930	2723	2.54	0.84 (0.67–1.06)
50–59	7378	2894	2.55	0.82 (0.65–1.04)
60+	8673	3300	2.63	0.87 (0.68–1.11)
Median overall length of stay				
< 1 year	7041	2653	2.65	Ref.
1–2 years	18,604	6952	2.68	1.02 (0.84–1.25)
> 2 years	3044	1126	2.70	1.04 (0.77–1.41)
Clinical staff per 100 residents				
< 10	6941	2643	2.63	Ref.
10–19	15,143	5612	2.70	0.99 (0.81–1.22)
20+	6605	2476	2.67	1.00 (0.79–1.26)
Care staff per 100 residents				
< 60	7219	2554	2.83	Ref.
60–79	16,211	6187	2.62	0.89 (0.73–1.09)
80+	5259	1990	2.64	0.86 (0.67–1.11)
Percentage residents with dementia				
< 10	6987	2320	3.01	Ref.
10–80	18,411	7065	2.61	0.85 (0.70–1.03)
80–100	3291	1346	2.44	0.82 (0.62–1.07)
Percentage residents with nursing care				
< 10	3594	1351	2.66	Ref.
10–80	13,278	5055	2.63	0.89 (0.69–1.14)
80–100	11,817	4325	2.73	1.03 (0.80–1.33)
Number of infection incidents per bed per year				
Less than 1	16,329	6548	2.49	Ref.
1 to 2	8316	2887	2.88	1.14 (0.94–1.37)
2 or more	4044	1297	3.12	1.24 (0.97–1.59)

*BNF* British National Formulary, *CI* confidence interval, *Ref*. reference group

**Table 3:** Multivariable analysis of antibiotic prescribing

Variable	Adjusted incidence rate ratio (95% CI)
Gender	
Male	Ref.
Female	1.03 (0.98–1.08)
Age	
65–74	Ref.
75–84	0.96 (0.89–1.04)
85–94	0.99 (0.92–1.07)
95+	1.11 (0.99–1.24)
Type of care	
Residential	Ref.
Nursing	1.21 (1.13–1.30)
Dementia	
No	Ref.
Yes	0.94 (0.89–1.00)
Respite care	
No	Ref.
Yes	1.12 (1.0–1.24)
Entered care home during study period	
No	Ref.
Yes	1.37 (1.30–1.44)
Status at end of study period	
In home	Ref.
Permanently Discharged	1.44 (1.32–1.57)
Died	1.58 (1.50–1.67)
Number of reported infection episodes during study	
0	Ref.
1	1.44 (1.35–1.54)
More than 1	2.09 (1.96–2.24)
Number of BNF chapters with repeated prescriptions (excluding antibiotics)	
0–1	Ref.
2–4	1.94 (1.76–2.14)
5–7	2.38 (2.16–2.62)
8 or more	2.89 (2.54–3.28)

*BNF* British National Formulary, *CI* confidence interval; *Ref*. reference group

### Sensitivity analysis

In the sensitivity analysis based on residents who had at least one pharmacy record, 16,247 residents were included across 235 homes. The total follow-up time was 5,178,046 resident-days (14,186 resident-years). There were 39,809 antibiotic prescriptions, a crude rate of 2.81 antibiotic prescriptions per resident year (95% CI 2.78–2.84). Negative binomial regression models resulted in similar associations to the main analysis. Full results of sensitivity analyses are shown in Additional File 4.

## Discussion

In this study of linked administrative and pharmacy data, we have demonstrated high rates of antibiotic prescribing for care home residents with large variation by home. There were clear associations between higher prescribing and resident factors, but we found no significant associations between prescribing rates and care home-level characteristics. We estimated that 30% of all antibiotic prescriptions dispensed were repeat prescriptions.

This was the first large-scale study in the UK to estimate rates of antibiotic use for care home residents. Our estimate of 2.68 antibiotic prescriptions per resident year is comparable to to estimates from smaller studies of care home residents in Hampshire, England (1.99 per resident year) [10] and South Wales (2.16 per resident year) [30]**.** These estimates are higher than rates for older adults in the general population in England derived from primary care data, which have been estimated at 1.06 per year for those aged 65–84, [17] 1.50 per year for those aged 85 and over, [17] and 1.13 per year for all those aged over 65 [18]. There are few published comparable European or other international estimates from care home settings. A USA study in 2001–2002 reported a rate of 1.75 prescriptions per resident year [31]; a study in British Columbia, Canada in 2007–2014 reported 35–39 defined daily doses of antibiotics per 1000 resident days [32]**,** and a study in Ontario, Canada reported 55 antibiotic days per 1000 resident days [33]**.** As our study measured rates of prescribing, and we lacked reliable information on the duration of therapy for each dispensed antibiotic, our estimates are not directly comparable to the HALT point prevalence surveys [12–14].

We found that higher rates of antibiotic prescribing were associated with residents who were likely to be more unwell, including those who had more infections, more probable co-morbidities (defined by repeated prescriptions from more BNF chapters), and those who died or permanently moved out of the home (likely to hospital) during the study period. There was also an association between higher antibiotic prescribing and residents who had recently moved into a home. Assuming that moving into a home is often linked to adverse health events, this association is also likely to represent residents in more ill health. We found variation in antibiotic prescribing rates between care homes, but this was not explained by the care home-level factors that we were able to investigate using routinely-collected data. This variation indicates scope for improvement in prescribing, and further investigation is warranted to explore the importance of other individual and contextual factors that we were not able to measure. Previous studies in care homes have identified a range of factors that may influence prescribing behaviour including past tendency of the physician to prescribe antibiotics [34, 35]**,** presence of an antimicrobial stewardship committee [36]**,** and practices around the use of urinary catheters [36]**.**

We also found that a high proportion of the antibiotics used (30%) were likely to be repeat prescriptions. The antibiotics that were most frequently repeated for long durations were azithromycin, cephalexin, nitrofurantoin and trimethoprim. These antibiotics are recommended for use as prophylaxis for chronic obstructive pulmonary disease and urinary tract infections [37, 38]. Long-term prophylactic use of antibiotics may therefore represent an important opportunity for improving antibiotic stewardship in care homes, particularly given that there is limited evidence that using antibiotics as prophylaxis is beneficial in this setting [39].

Our findings have implications for antimicrobial stewardship intervention design and implementation. Reviews of existing interventions in care homes have found few high quality studies [40–43], and the behaviours targeted by interventions are often poorly specified. We have identified two possible targets for future behavioural interventions: reducing long term prophylactic prescriptions and optimising antibiotic use for residents who have recently moved into a home or are near the end of life. Our findings also highlight the need to investigate other behaviours related to stewardship that precede an antibiotic being prescribed such as identifying, diagnosing, escalating, and managing suspected infections. Safely reducing antibiotic use in care homes could be achieved by improved infection prevention and control measures such as vaccination, isolation of symptomatic residents, handwashing, exclusion of symptomatic visitors, improved catheter management, regular movement, and good skin care [44].

A strength of this study was its large scale and use of novel linkages between care home administrative data and pharmacy drug dispensing data. This allowed us to explore relationships between antibiotic prescribing rates and resident and care home characteristics at scale for the first time in the UK. As the majority of other estimates of antibiotic use in care homes in the UK have been based on point prevalence data or small data sets, our results are more representative of the population and less likely to be affected by seasonal variation.

A limitation of this study was that, although the pharmacy chain is contracted to provide all prescriptions to the care home chain, the data suggest that this does not always happen in practice. To account for this, we conducted two analyses with different inclusion criteria. The main analysis included all residents from care homes from which at least 75% of residents matched to pharmacy data, and the sensitivity analysis included all residents (from any care home) that matched to at least one pharmacy record. As expected, the sensitivity analysis produced a slightly higher rate (2.81 antibiotic prescriptions per resident year compared to 2.61 in the main analysis), as this analysis excluded any residents who did not have any prescriptions. However, both analyses may still be underestimates of prescribing if many antibiotics were dispensed from different pharmacies. Characteristics of residents and care homes included and excluded from the main analysis (additional file 2) were similar, suggesting that factors associated with increased prescribing are not due to bias in the data. Antibiotics dispensed during hospital stays were also not captured in this study.

Another limitation was that our analysis used data from administrative care home systems that were not designed for research. Information on temporary absences from homes, for example during hospital stays, or temporary stays in homes funded by the local authority were not available. Although we found increased antibiotic prescribing for residents with more probable co-morbidities, this was based on a crude measure of repeat prescriptions of non-antibiotic drugs. We did not have information on specific co-morbidities or other medical risk factors such as catheter use. Since the pharmacy data does not include indication for the drug dispensed, we were also not able to directly assess the appropriateness of the antibiotic prescriptions. However, we performed exploratory analyses examining how well the antibiotic prescriptions matched the broad category of infection reported in the care home incident monitoring systems. This showed that, in general, antibiotics were of expected classes for a given infection category. Further work is needed to investigate prescribing patterns for specific types of infection with or without microbiological confirmation. Improved recording of care home residency in primary and secondary care records, and enhanced data collection within care homes, would enable these factors to be assessed in greater detail.

## Conclusions

In summary, this large-scale study has demonstrated high rates of antibiotic use for residents of care homes in the UK and a high degree of variation across homes. Although antibiotics were mainly used for the most unwell residents, the high variation in antibiotic use suggests scope for improved stewardship. Our analysis has identified potential targets for future stewardship interventions, but further work is needed to characterise the drivers of prescribing in care homes to inform the development of interventions that target the influences of prescribing.

## Supplementary information

**Additional file 1:** Distribution of time between prescriptions of the same antibiotic for the same resident.

**Additional file 2.** Characteristics of residents and care homes included and excluded from main analysis

**Additional file 3: **Country-specific analyses of rates of antibiotic prescribing in care homes **Table 1.** Care home **Table 2**. Rates and single variable negative binomial regressioncharacteristics **Table 3**. Multivariable negative binomial regression

**Additional file 4: **Sensitivity analysis **Table 1**. Resident and care home characteristics **Table 2**. Rates and single variable analysis of antibiotic prescribing by resident and care home characteristics **Table 3**. Multivariable analysis of antibiotic prescribing

## Data Availability

The datasets analysed during the current study are not publicly available as they were used through agreement with the care home provider (Four Seasons Health Care) and pharmacy (Boots UK). Data are however available from the authors upon reasonable request and with permission of Four Seasons Health Care and Boots UK.

## Abbreviations

aIRR: Adjusted Incidence Rate Ratio
ATC: Anatomical Therapeutic Chemical
BNF: British National Formulary
CI: Confidence interval
CQC: Care Quality Commission
ECDC: European Centre for Disease Prevention and Control
HALT: Healthcare-associated infections in long-term care facilities
IQR: Interquartile range
