# Supplementary figures and images for: Antibiotic prescribing in UK care homes 2016–2017: retrospective cohort study of linked data
Source: BMC Health Serv Res. 2020 Jun 18;20:555. doi: 10.1186/s12913-020-05422-z (PMC7301534; doi:10.1186/s12913-020-05422-z)

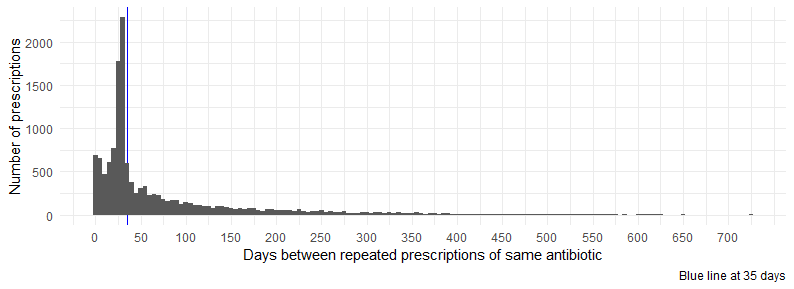

Supplement: Supplementary file 1 — Additional file 1: Distribution of time between prescriptions of the same antibiotic for the same resident. [file 12913_2020_5422_MOESM1_ESM.png]
