# Supplementary material for: Antibiotic prescribing in UK care homes 2016–2017: retrospective cohort study of linked data
Source: BMC Health Serv Res. 2020 Jun 18;20:555. doi: 10.1186/s12913-020-05422-z (PMC7301534; doi:10.1186/s12913-020-05422-z)
Supplement: Supplementary file 2 — Additional file 2. Characteristics of residents and care homes included and excluded from main analysis [file 12913_2020_5422_MOESM2_ESM.docx]

# Additional file 2: Characteristics of residents and care homes included and excluded from main analysis

|  |  | **Main analysis** | | **Excluded care homes** | |
| --- | --- | --- | --- | --- | --- |
| **Variable** | **Value** | **Number** | **Percentage** | **Number** | **Percentage** |
| **Resident-level** | **Total** | **13487** |  | **11,996** |  |
| Gender | Female | 8518 | 63.16 | 7587 | 63.25 |
|  | Male | 4969 | 36.84 | 4409 | 36.75 |
| Age | 65-74 | 1871 | 13.87 | 1888 | 15.74 |
|  | 75-84 | 5049 | 37.44 | 4394 | 36.63 |
|  | 85-94 | 5691 | 42.20 | 4993 | 41.62 |
|  | 95+ | 876 | 6.50 | 721 | 6.01 |
| Type of care | Residential | 4354 | 32.28 | 3302 | 27.53 |
|  | Nursing | 9109 | 67.54 | 8626 | 71.91 |
| Dementia | No | 8246 | 61.14 | 7966 | 66.41 |
|  | Yes | 5217 | 38.68 | 3962 | 33.03 |
| Respite care | No | 11459 | 84.96 | 9575 | 79.82 |
|  | Yes | 2028 | 15.04 | 2421 | 20.18 |
| Length of stay during study period | Median, IQR | 210 | 51-509 | 194 | 44-483 |
| Overall length of stay | Median, IQR | 333 | 67-913 | 304 | 55-910 |
| Entered care home during study period | No | 5587 | 41.43 | 4942 | 41.20 |
|  | Yes | 7900 | 58.57 | 7054 | 58.80 |
| Status at end of study period | In home | 3772 | 27.97 | 3077 | 25.65 |
|  | Permanently Discharged | 2688 | 19.93 | 3106 | 25.89 |
|  | Died | 7027 | 52.10 | 5813 | 48.46 |
| Number of infection episodes during study | 0 | 8993 | 66.68 | 7599 | 63.35 |
|  | 1 | 2217 | 16.44 | 1735 | 14.46 |
|  | More than 1 | 2277 | 16.88 | 2662 | 22.19 |
| **Care home-level** | **Total** | **135** |  | **124** |  |
| Country | England | 93 | 68.89 | 67 | 54.47 |
|  | Northern Ireland | 20 | 14.82 | 38 | 30.89 |
|  | Scotland | 19 | 14.07 | 14 | 11.38 |
|  | Wales | 3 | 2.22 | 4 | 3.25 |
| Number of beds | <40 | 34 | 25.19 | 37 | 30.08 |
|  | 40-49 | 37 | 27.41 | 28 | 22.76 |
|  | 50-59 | 34 | 25.19 | 24 | 19.51 |
|  | 60+ | 30 | 22.22 | 34 | 27.64 |
| Median overall length of stay | <1 year | 34 | 25.19 | 47 | 38.21 |
|  | 1-2 years | 85 | 62.96 | 63 | 51.22 |
|  | >2 years | 16 | 11.85 | 13 | 10.57 |
| Clinical staff per 100 residents | <10 | 35 | 25.93 | 29 | 23.58 |
|  | 10-19 | 65 | 48.15 | 45 | 36.59 |
|  | 20+ | 35 | 25.93 | 47 | 38.21 |
| Care staff per 100 residents | <60 | 33 | 24.44 | 27 | 21.95 |
|  | 60-79 | 75 | 55.56 | 67 | 54.47 |
|  | 80+ | 27 | 20.00 | 28 | 22.76 |
| Percentage residents with dementia | <10 | 35 | 25.93 | 37 | 30.08 |
|  | 10-80 | 80 | 59.26 | 70 | 56.91 |
|  | 80-100 | 20 | 14.82 | 16 | 13.01 |
| Percentage residents with nursing care | <10 | 20 | 14.82 | 15 | 12.20 |
|  | 10-80 | 58 | 42.96 | 41 | 33.33 |
|  | 80-100 | 57 | 42.22 | 67 | 54.47 |
| Number of infection incidents per bed per year | Less than 1 | 70 | 51.90 | 65 | 52.85 |
|  | 1 to 2 | 39 | 28.90 | 38 | 30.89 |
|  | 2 or more | 26 | 19.30 | 19 | 15.45 |
| **Care home-level, England only** | **Total** | **93** |  | 67 |  |
| Overall CQC rating | Room for improvement | 36 | 26.67 | 25 | 20.33 |
|  | Good | 55 | 40.74 | 41 | 33.33 |
|  | Outstanding | 2 | 1.48 |  |  |
| Deprivation decile | Median, IQR | 4 | 3-6 | 5 | 2-7 |
| Urban/Rural | Urban | 84 | 90.32 | 56 | 83.58 |
|  | Mixed | 6 | 6.45 | 9 | 13.43 |
|  | Rural | 3 | 3.23 | 2 | 2.99 |
| **Care home-level, Northern Ireland only** | **Total** | **20** |  | 38 |  |
| Deprivation decile | Median, IQR | 7.5 | 5.75-9 | 8 | 7-8.75 |
| Urban/Rural | Urban | 17 | 85 | 30 | 78.95 |
|  | Mixed | 1 | 5 | 3 | 7.89 |
|  | Rural | 2 | 10 | 5 | 13.16 |
| **Care home-level, Scotland only** | **Total** | **19** |  | 14 |  |
| Deprivation decile | Median, IQR | 5 | 3-7 | 7 | 4.25-8.5 |
| Urban/Rural | Urban | 19 | 100 | 12 | 85.71 |
|  | Mixed | 0 | 0 | 1 | 7.14 |
|  | Rural | 0 | 0 | 1 | 7.14 |
| **Care home-level, Wales only** | **Total** | **3** |  | 4 |  |
| Deprivation decile | Median, IQR | 8 | 6-9 | 2 | 1-3 |
| Urban/Rural | Urban | 1 | 33.33 | 4 | 100 |
|  | Mixed | 2 | 66.67 |  |  |

Where numbers do not sum to total residents/care homes this is due to missing data
