# Supplementary material for: Antibiotic prescribing in UK care homes 2016–2017: retrospective cohort study of linked data
Source: BMC Health Serv Res. 2020 Jun 18;20:555. doi: 10.1186/s12913-020-05422-z (PMC7301534; doi:10.1186/s12913-020-05422-z)
Supplement: Supplementary file 3 — Additional file 3: Country-specific analyses of rates of antibiotic prescribing in care homes Table 1. Care home Table 2. Rates and single variable negative binomial regressioncharacteristics Table 3. Multivariable negative binomial regression [file 12913_2020_5422_MOESM3_ESM.docx]

# Additional file 3: Country-specific analyses of rates of antibiotic prescribing in care homes

## Table 1: Care home characteristics

| **England only (n=93)** | **Number** | **Percentage** |
| --- | --- | --- |
| Overall CQC rating |  |  |
| Requires improvement | 36 | 26.7 |
| Good | 55 | 40.7 |
| Outstanding | 2 | 1.5 |
| Deprivation decile |  |  |
| Median (IQR) | 4 | 3-6 |
| Urban/Rural |  |  |
| Urban | 84 | 90.3 |
| Mixed | 6 | 6.5 |
| Rural | 3 | 3.2 |
| **Northern Ireland only (n=20)** |  |  |
| Deprivation decile |  |  |
| Median (IQR) | 7.5 | 5.75-9 |
| Urban/Rural |  |  |
| Urban | 17 | 85.0 |
| Mixed | 1 | 5.0 |
| Rural | 2 | 10.0 |
| **Scotland only (n=19)** |  |  |
| Deprivation decile |  |  |
| Median (IQR) | 5 | 3-7 |
| Urban/Rural |  |  |
| Urban | 19 | 100 |
| **Wales only (n=3)** |  |  |
| Deprivation decile |  |  |
| Median (IQR) | 8 | 6-9 |
| Urban/Rural |  |  |
| Urban | 1 | 33.3 |
| Mixed | 2 | 66.7 |

## Table 2: Rates and single variable negative binomial regression

| **Variable** | **Value** | **Number of antibiotic prescriptions** | **Resident years** | **Antibiotic prescriptions per resident year** | **Incidence rate ratio (95% CI)** |
| --- | --- | --- | --- | --- | --- |
| **England only** | |  |  |  |  |
| Overall CQC rating | Requires improvement | 8,133 | 3,078 | 2.64 | Ref. |
|  | Good | 10,812 | 4,196 | 2.58 | 0.95 (0.75-1.19) |
|  | Outstanding | 261 | 168 | 1.56 | 0.59 (0.27-1.30) |
| Deprivation decile | Overall estimate |  |  |  | 1.03 (0.98-1.08) |
|  | 1 | 1,750 | 702 | 2.49 |  |
|  | 2 | 2,342 | 1,151 | 2.03 |  |
|  | 3 | 3,085 | 1,088 | 2.84 |  |
|  | 4 | 2,495 | 1,054 | 2.37 |  |
|  | 5 | 2,429 | 1,037 | 2.34 |  |
|  | 6 | 3,984 | 1,324 | 3.01 |  |
|  | 7 | 563 | 220 | 2.56 |  |
|  | 8 | 1,083 | 455 | 2.38 |  |
|  | 9 | 1,129 | 320 | 3.53 |  |
|  | 10 | 346 | 91 | 3.79 |  |
| Urban/Rural | Urban | 17,259 | 6,812 | 2.53 | Ref. |
|  | Mixed | 1,237 | 432 | 2.87 | 1.15 (0.73-1.82) |
|  | Rural | 710 | 197 | 3.60 | 1.51 (0.80-2.85) |
| **Northern Ireland only** | |  |  |  |  |
| Deprivation decile | Overall estimate |  |  |  | 1.01 (0.96-1.06) |
|  | 4 | 1,148 | 312 | 3.68 |  |
|  | 5 | 176 | 84 | 2.09 |  |
|  | 6 | 810 | 279 | 2.90 |  |
|  | 7 | 129 | 53 | 2.42 |  |
|  | 8 | 554 | 174 | 3.18 |  |
|  | 9 | 771 | 288 | 2.67 |  |
|  | 10 | 383 | 122 | 3.13 |  |
| Urban/Rural | Urban | 3,491 | 1151 | 3.03 | Ref. |
|  | Mixed | 204 | 65 | 3.16 | 1.10 (0.68-1.78) |
|  | Rural | 276 | 97 | 2.83 | 0.89 (0.61-1.30) |
| **Scotland only** | |  |  |  |  |
| Deprivation decile |  |  |  |  | 0.95 (0.90-1.00) |
|  | 1 | 411 | 156 | 2.64 |  |
|  | 2 | 585 | 203 | 2.88 |  |
|  | 3 | 807 | 285 | 2.83 |  |
|  | 4 | 310 | 143 | 2.17 |  |
|  | 5 | 774 | 255 | 3.04 |  |
|  | 7 | 1,147 | 409 | 2.81 |  |
|  | 8 | 152 | 110 | 1.38 |  |
|  | 9 | 205 | 97 | 2.11 |  |
|  | 10 | 135 | 69 | 1.96 |  |
| Urban/Rural | Urban | 4,526 | 1726 | 2.62 | - |
| **Wales only** | |  |  |  |  |
| Deprivation decile |  |  |  |  | 1.00 (0.95-1.06) |
|  | 4 | 312 | 77 | 4.03 |  |
|  | 8 | 492 | 127 | 3.87 |  |
|  | 10 | 182 | 47 | 3.90 |  |
| Urban/Rural | Urban | 312 | 77 | 4.03 | Ref. |
|  | Mixed | 674 | 174 | 3.88 | 0.94 (0.72-1.24) |

CI, confidence interval; CQC Care Quality Commission; Ref., reference group

## Table 3: Multivariable negative binomial regression

### England only

| **Variable** | **Value** | | **Adjusted incidence rate ratio (95% CI)** |  |
| --- | --- | --- | --- | --- |
| **England only** |  | |  |  |
| Resident-level |  | |  |  |
| Gender | Male | |  |  |
|  | Female | | 0.97 (0.92-1.03) |  |
| Age | 65-74 | |  |  |
|  | 75-84 | | 0.97 (0.89-1.07) |  |
|  | 85-94 | | 0.98 (0.90-1.08) |  |
|  | 95+ | | 1.17 (1.02-1.34) |  |
| Type of care | Residential | |  |  |
|  | Nursing | | 1.17 (1.09-1.26) |  |
| Dementia | No | |  |  |
|  | Yes | | 0.89 (0.83-0.96) |  |
| Respite care | No | |  |  |
|  | Yes | | 1.03 (0.92-1.16) |  |
| Entered care home during study period | No | |  |  |
|  | Yes | | 1.41 (1.33-1.50) |  |
| Status at end of study period | In home | |  |  |
|  | Permanently Discharged | | 1.36 (1.22-1.51) |  |
|  | Died | | 1.56 (1.46-1.66) |  |
| Number of infection episodes during study | 0 | |  |  |
|  | 1 | | 1.32 (1.23-1.43) |  |
|  | More than 1 | | 1.92 (1.76-2.08) |  |
| Number of BNF chapters with repeated prescriptions (excluding antibiotics) | 0-1 | |  |  |
|  | 2-4 | | 1.89 (1.70-2.09) |  |
|  | 5-7 | | 2.39 (2.15-2.64) |  |
|  | 8 or more | | 3.12 (2.69-3.61) |  |
| Care home-level |  | |  |  |
| Overall CQC rating | Requires improvement | |  |  |
|  | Good | | 1.02 (0.81-1.29) |  |
|  | Outstanding | | 1.02 (0.46-2.25) |  |
| Deprivation decile | Overall estimate | | 1.02 (0.97-1.07) |  |
| **Northern Ireland only** | |  |  | |
| Resident-level | |  |  | |
| Gender | | Male |  | |
|  | | Female | 1.24 (1.06-1.44) | |
| Age | | 65-74 |  | |
|  | | 75-84 | 0.94 (0.76-1.15) | |
|  | | 85-94 | 0.95 (0.77-1.17) | |
|  | | 95+ | 0.68 (0.49-0.95) | |
| Type of care | | Residential |  | |
|  | | Nursing | 1.33 (1.01-1.75) | |
| Dementia | | No |  | |
|  | | Yes | 0.89 (0.75-1.04) | |
| Respite care | | No |  | |
|  | | Yes | 1.48 (1.14-1.94) | |
| Entered care home during study period | | No |  | |
|  | | Yes | 1.34 (1.16-1.56) | |
| Status at end of study period | | In home |  | |
|  | | Permanently Discharged | 1.59 (1.27-1.99) | |
|  | | Died | 1.57 (1.34-1.84) | |
| Number of infection episodes during study | | 0 |  | |
|  | | 1 | 2.21 (1.79-2.73) | |
|  | | More than 1 | 3.13 (2.60-3.77) | |
| Number of BNF chapters with repeated prescriptions (excluding antibiotics) | | 0-1 |  | |
|  | | 2-4 | 3.07 (2.31-4.07) | |
|  | | 5-7 | 3.75 (2.87-4.91) | |
|  | | 8 or more | 3.56 (2.59-4.89) | |
| Care home-level | |  |  | |
| Deprivation decile | | Overall estimate | 1.02 (0.96-1.08) | |
| **Scotland only** | |  |  | |
| Resident-level | |  |  | |
| Gender | | Male |  | |
|  | | Female | 1.13 (0.99-1.29) | |
| Age | | 65-74 |  | |
|  | | 75-84 | 0.94 (0.79-1.12) | |
|  | | 85-94 | 1.01 (0.84-1.21) | |
|  | | 95+ | 1.22 (0.90-1.64) | |
| Type of care | | Residential |  | |
|  | | Nursing | 1.25 (0.96-1.63) | |
| Dementia | | No |  | |
|  | | Yes | 1.13 (0.99-1.29) | |
| Respite care | | No |  | |
|  | | Yes | 1.43 (0.90-2.27) | |
| Entered care home during study period | | No |  | |
|  | | Yes | 1.18 (1.05-1.34) | |
| Status at end of study period | | In home |  | |
|  | | Permanently Discharged | 1.67 (1.30-2.15) | |
|  | | Died | 1.61 (1.42-1.84) | |
| Number of infection episodes during study | | 0 |  | |
|  | | 1 | 1.60 (1.37-1.87) | |
|  | | More than 1 | 2.39 (2.06-2.77) | |
| Number of BNF chapters with repeated prescriptions (excluding antibiotics) | | 0-1 |  | |
|  | | 2-4 | 1.58 (1.17-2.12) | |
|  | | 5-7 | 1.72 (1.28-2.32) | |
|  | | 8 or more | 1.87 (1.29-2.69) | |
| Care home-level | |  |  | |
| Deprivation decile | | Overall estimate | 0.95 (0.90-1.00) | |
| **Wales only** |  | |  | |
| Resident-level |  | |  | |
| Gender | Male | |  | |
|  | Female | | 1.21 (0.91-1.60) | |
| Age | 65-74 | |  | |
|  | 75-84 | | 1.03 (0.66-1.61) | |
|  | 85-94 | | 1.26 (0.82-1.95) | |
|  | 95+ | | 1.16 (0.63-2.12) | |
| Type of care | Residential | |  | |
|  | Nursing | | 1.38 (0.98-1.93) | |
| Dementia | No | |  | |
|  | Yes | | 0.97 (0.69-1.37) | |
| Respite care | No | |  | |
|  | Yes | | 1.20 (0.35-4.07) | |
| Entered care home during study period | No | |  | |
|  | Yes | | 1.58 (1.21-2.05) | |
| Status at end of study period | In home | |  | |
|  | Permanently Discharged | | 0.99 (0.55-1.78) | |
|  | Died | | 1.42 (1.07-1.87) | |
| Number of infection episodes during study | 0 | |  | |
|  | 1 | | 1.69 (1.14-2.50) | |
|  | More than 1 | | 2.18 (1.52-3.11) | |
| Number of BNF chapters with repeated prescriptions (excluding antibiotics) | 0-1 | |  | |
|  | 2-4 | | 0.83 (0.45-1.53) | |
|  | 5-7 | | 0.93 (0.50-1.72) | |
|  | 8 or more | | 1.54 (0.72-3.30) | |
| Care home-level |  | |  | |
| Deprivation decile | Overall estimate | | 0.96 (0.91-1.01) | |

BNF, British National Formulary; CI, confidence interval; CQC Care Quality Commission
