# Supplementary material for: Antibiotic prescribing in UK care homes 2016–2017: retrospective cohort study of linked data
Source: BMC Health Serv Res. 2020 Jun 18;20:555. doi: 10.1186/s12913-020-05422-z (PMC7301534; doi:10.1186/s12913-020-05422-z)
Supplement: Supplementary file 4 — Additional file 4: Sensitivity analysis Table 1. Resident and care home characteristics Table 2. Rates and single variable analysis of antibiotic prescribing by resident and care home characteristics Table 3. Multivariable analysis of antibiotic prescribing [file 12913_2020_5422_MOESM4_ESM.docx]

# Additional file 4: Sensitivity analysis

## Table 1: Resident and care home characteristics

| **Variable** | **Number** | **Percentage** |
| --- | --- | --- |
| **Resident-level (n=16,427)** |  |  |
| Gender |  |  |
| Male | 10,423 | 64.15 |
| Female | 5,824 | 35.85 |
| Age |  |  |
| 65-74 | 2,286 | 14.07 |
| 75-84 | 6,059 | 37.29 |
| 85-94 | 6,898 | 42.46 |
| 95+ | 1,004 | 6.18 |
| Type of care |  |  |
| Residential | 11,226 | 69.10 |
| Nursing | 4,979 | 30.65 |
| Dementia |  |  |
| No | 10,121 | 62.30 |
| Yes | 6,084 | 37.45 |
| Respite care |  |  |
| No | 14,439 | 88.87 |
| Yes | 1,808 | 11.13 |
| Length of stay during study period (days)  Median (IQR) | 255 | 80-549 |
| Overall length of stay (days)  Median (IQR) | 394 | 105-992 |
| Entered care home during study period |  |  |
| No | 7,242 | 44.40 |
| Yes | 9,069 | 55.60 |
| Status at end of study period |  |  |
| In home | 8,662 | 53.31 |
| Permanently Discharged | 5,074 | 31.23 |
| Died | 2,511 | 15.46 |
| Number of reported infection episodes during study |  |  |
| 0 | 10,122 | 62.06 |
| 1 | 2,916 | 17.88 |
| More than 1 | 3,273 | 20.07 |
| Number of BNF chapters with repeated prescriptions (excluding antibiotics) |  |  |
| 0-1 |  |  |
| 2-4 |  |  |
| 5-7 |  |  |
| 8 or more |  |  |
| **Care home-level (n=235)** |  |  |
| Country |  |  |
| England | 148 | 62.98 |
| Northern Ireland | 49 | 20.85 |
| Scotland | 31 | 13.19 |
| Wales | 7 | 2.98 |
| Number of beds |  |  |
| <40 | 2,869 | 17.66 |
| 40-49 | 6,035 | 37.15 |
| 50-59 | 6,515 | 40.10 |
| 60+ | 828 | 5.10 |
| Median overall length of stay |  |  |
| <1 year | 65 | 27.66 |
| 1-2 years | 137 | 58.30 |
| >2 years | 33 | 14.04 |
| Clinical staff per 100 residents |  |  |
| <10 | 68 | 28.94 |
| 10-19 | 140 | 59.57 |
| 20+ | 27 | 11.49 |
| Care staff per 100 residents |  |  |
| <60 | 33 | 14.04 |
| 60-79 | 93 | 39.57 |
| 80+ | 109 | 46.38 |
| Percentage residents with dementia |  |  |
| <10 | 137 | 58.30 |
| 10-80 | 66 | 28.09 |
| 80-100 | 32 | 13.62 |
| Percentage residents with nursing care |  |  |
| <10 | 60 | 25.53 |
| 10-80 | 104 | 44.26 |
| 80-100 | 70 | 29.79 |
| Number of infection incidents per bed per year |  |  |
| Less than 1 | 56 | 23.83 |
| 1 to 2 | 129 | 54.89 |
| 2 or more | 50 | 21.28 |
| **Care home-level, England only (n=148)** |  |  |
| Overall CQC rating |  |  |
| Requires improvement | 56 | 23.83 |
| Good | 90 | 38.30 |
| Outstanding | 2 | 0.85 |
| Deprivation decile |  |  |
| Median (IQR) | 4 | 2-6 |
| Urban/Rural |  |  |
| Urban | 130 | 87.84 |
| Mixed | 14 | 9.46 |
| Rural | 4 | 2.7 |
| **Care home-level, Northern Ireland only (n=49)** |  |  |
| Deprivation decile |  |  |
| Median (IQR) | 8 | 5-9 |
| Urban/Rural |  |  |
| Urban | 39 | 79.59 |
| Mixed | 4 | 8.16 |
| Rural | 6 | 12.24 |
| **Care home-level, Scotland only (n=31)** |  |  |
| Deprivation decile |  |  |
| Median (IQR) | 6 | 3-7 |
| Urban/Rural |  |  |
| Urban | 29 | 93.55 |
| Mixed | 1 | 3.23 |
| Rural | 1 | 3.23 |
| **Care home-level, Wales only (n=7)** |  |  |
| Deprivation decile |  |  |
| Median (IQR) | 3 | 2-6 |
| Urban/Rural |  |  |
| Urban | 5 | 71.43 |
| Mixed | 2 | 28.57 |

## Table 2: Rates and single variable analysis of antibiotic prescribing by resident and care home characteristics

| **Variable** | **N antibiotic prescriptions** | **Resident years** | **Antibiotic prescriptions per resident year** | **Incidence rate ratio (95% CI)** |
| --- | --- | --- | --- | --- |
| **Resident-level** |  |  |  |  |
| Gender | 12,854 | 4652 | 2.76 |  |
| Male |  |  |  |  |
| Female | 27,123 | 9614 | 2.82 | 0.97 (0.93-1.02) |
| Age | 5,442 | 2066 | 2.63 |  |
| 65-74 |  |  |  |  |
| 75-84 | 13,975 | 5252 | 2.66 | 1.03 (0.96-1.10) |
| 85-94 | 17,773 | 6042 | 2.94 | 1.11 (1.04-1.18) |
| 95+ | 2,787 | 906 | 3.08 | 1.17 (1.06-1.29) |
| Type of care | 11,345 | 4528 | 2.51 |  |
| Residential |  |  |  |  |
| Nursing | 28,486 | 9680 | 2.94 | 1.23 (1.16-1.31) |
| Dementia | 24,726 | 8291 | 2.98 |  |
| No |  |  |  |  |
| Yes | 15,105 | 5916 | 2.55 | 0.87 (0.82-0.91) |
| Respite care | 38,241 | 13639 | 2.80 |  |
| No |  |  |  |  |
| Yes | 1,736 | 626 | 2.77 | 1.08 (0.99-1.17) |
| Entered care home during study period | 24,166 | 9048 | 2.67 |  |
| No |  |  |  |  |
| Yes | 15,811 | 5218 | 3.03 | 1.15 (1.11-1.20) |
| Status at end of study period | 14,696 | 6243 | 2.35 |  |
| In home |  |  |  |  |
| Permanently Discharged | 3,203 | 1091 | 2.94 | 1.32 (1.23-1.42) |
| Died | 22,078 | 6931 | 3.19 | 1.48 (1.42-1.55) |
| Number of infection episodes during study | 14,409 | 6972 | 2.07 |  |
| 0 |  |  |  |  |
| 1 | 7,421 | 2876 | 2.58 | 1.40 (1.32-1.49) |
| More than 1 | 18,147 | 4417 | 4.11 | 2.03 (1.91-2.14) |
| Number of BNF chapters with repeated prescriptions (excluding antibiotics) | 1,215 | 624 | 1.95 |  |
| 0-1 |  |  |  |  |
| 2-4 | 11,001 | 5254 | 2.09 | 0.78 (0.71-0.85) |
| 5-7 | 22,944 | 7257 | 3.16 | 1.02 (0.94-1.11) |
| 8 or more | 4,817 | 1130 | 4.26 | 1.26 (1.13-1.41) |
| **Care home-level** |  |  |  |  |
| Country | 24,933 | 9448 | 2.64 |  |
| England |  |  |  |  |
| Northern Ireland | 7,731 | 2277 | 3.40 | 1.28 (1.03-1.58) |
| Scotland | 5,730 | 2169 | 2.64 | 0.99 (0.77-1.27) |
| Wales | 1,583 | 372 | 4.26 | 1.83 (1.12-2.99) |
| Number of beds | 7,686 | 2469 | 3.11 |  |
| <40 |  |  |  |  |
| 40-49 | 9,552 | 3574 | 2.67 | 0.91 (0.72-1.16) |
| 50-59 | 9,838 | 3626 | 2.71 | 0.99 (0.77-1.27) |
| 60+ | 12,901 | 4596 | 2.81 | 1.03 (0.81-1.31) |
| Median overall length of stay | 11,255 | 4090 | 2.75 |  |
| <1 year |  |  |  |  |
| 1-2 years | 24,844 | 8835 | 2.81 | 0.95 (0.79-1.15) |
| >2 years | 3,878 | 1340 | 2.89 | 0.97 (0.71-1.32) |
| Clinical staff per 100 residents | 9,484 | 3533 | 2.68 |  |
| <10 |  |  |  |  |
| 10-19 | 20,881 | 7220 | 2.89 | 1.14 (0.92-1.41) |
| 20+ | 9,540 | 3488 | 2.74 | 0.96 (0.76-1.21) |
| Care staff per 100 residents | 9,841 | 3232 | 3.04 |  |
| <60 |  |  |  |  |
| 60-79 | 22,649 | 8339 | 2.72 | 0.86 (0.70-1.06) |
| 80+ | 7,487 | 2694 | 2.78 | 0.82 (0.64-1.06) |
| Percentage residents with dementia | 10,016 | 3231 | 3.10 |  |
| <10 |  |  |  |  |
| 10-80 | 25,402 | 9369 | 2.71 | 0.91 (0.75-1.11) |
| 80-100 | 4,559 | 1666 | 2.74 | 0.90 (0.67-1.19) |
| Percentage residents with nursing care | 4,813 | 1787 | 2.69 |  |
| <10 |  |  |  |  |
| 10-80 | 16,694 | 6304 | 2.65 | 0.97 (0.75-1.26) |
| 80-100 | 18,470 | 6174 | 2.99 | 1.23 (0.95-1.60) |
| Number of infection incidents per bed per year | 22,419 | 8559 | 2.62 |  |
| Less than 1 |  |  |  |  |
| 1-2 | 11,862 | 3905 | 3.04 | 1.22 (1.00-1.48) |
| 2 or more | 5,696 | 1801 | 3.16 | 1.21 (0.94-1.56) |

BNF, British National Formulary; CI, confidence interval

## Table 3: Multivariable analysis of antibiotic prescribing

| **Variable** | **Adjusted incidence rate ratio (95% CI** |
| --- | --- |
| Gender |  |
| Male |  |
| Female | 1.01 (0.96-1.05) |
| Age |  |
| 65-74 |  |
| 75-84 | 0.98 (0.92-1.04) |
| 85-94 | 1.03 (0.96-1.09) |
| 95+ | 1.09 (0.99-1.20) |
| Type of care |  |
| Residential |  |
| Nursing | 1.20 (1.13-1.27) |
| Dementia |  |
| No |  |
| Yes | 0.95 (0.90-1.00) |
| Respite care |  |
| No |  |
| Yes | 1.07 (0.98-1.17) |
| Entered care home during study period |  |
| No |  |
| Yes | 1.33 (1.27-1.39) |
| Status at end of study period |  |
| In home |  |
| Permanently Discharged | 1.42 (1.32-1.53) |
| Died | 1.53 (1.46-1.60) |
| Number of infection episodes during study |  |
| 0 |  |
| 1 | 1.42 (1.34-1.50) |
| More than 1 | 2.10 (1.99-2.23) |
| Number of BNF chapters with repeated prescriptions (excluding antibiotics) |  |
| 0-1 |  |
| 2-4 | 0.77 (0.71-0.84) |
| 5-7 | 0.97 (0.89-1.06) |
| 8 or more | 1.16 (1.03-1.29) |

BNF, British National Formulary; CI, confidence interval
